# Time Perception and Depressive Realism: Judgment Type, Psychophysical Functions and Bias

**DOI:** 10.1371/journal.pone.0071585

**Published:** 2013-08-21

**Authors:** Diana E. Kornbrot, Rachel M. Msetfi, Melvyn J. Grimwood

**Affiliations:** 1 Psychology Department, University of Hertfordshire, Hertfordshire, United Kingdom; 2 Department of Psychology, Centre for Social Issues Research, University of Limerick, Limerick, Republic of Ireland; 3 Department of Psychology, University of Leicester, Leicester, United Kingdom; Duke University, United States of America

## Abstract

The effect of mild depression on time estimation and production was investigated. Participants made both magnitude estimation and magnitude production judgments for five time intervals (specified in seconds) from 3 sec to 65 sec. The parameters of the best fitting psychophysical function (power law exponent, intercept, and threshold) were determined individually for each participant in every condition. There were no significant effects of mood (high BDI, low BDI) or judgment (estimation, production) on the mean exponent, **n = .**98, 95% confidence interval (.96–1.04) or on the threshold. However, the intercept showed a ‘depressive realism’ effect, where high BDI participants had a smaller deviation from accuracy and a smaller difference between estimation and judgment than low BDI participants. Accuracy bias was assessed using three measures of accuracy: *difference*, defined as psychological time minus physical time, *ratio*, defined as psychological time divided by physical time, and a new logarithmic accuracy measure defined as *ln (ratio)*. The *ln (ratio)* measure was shown to have approximately normal residuals when subjected to a mixed ANOVA with mood as a between groups explanatory factor and judgment and time category as repeated measures explanatory factors. The residuals of the other two accuracy measures flagrantly violated normality. The mixed ANOVAs of accuracy also showed a strong depressive realism effect, just like the intercepts of the psychophysical functions. There was also a strong negative correlation between estimation and production judgments. Taken together these findings support a clock model of time estimation, combined with additional cognitive mechanisms to account for the depressive realism effect. The findings also suggest strong methodological recommendations.

## Introduction

Subjective time passes slowly for people who are in a depressed state [1], [2], [3], [4], [5], and they may use phrases such as ‘time seems to drag’ to describe their experiences However, people with depression are also reported to have more realistic perceptions in some cognitive tasks, labelled ‘depressive realism’ [3], [6], [7], [8]. Given this paradoxical combination of slowness and realism, it is not surprising then that evidence for mood effects on time perception has been inconsistent and contradictory. See [3] for a review.

Historically, there have been two main approaches to the psychology of direct time perception. The psychophysical judgment approach has used magnitude estimation and magnitude production tasks and has used estimated parameters of the psychophysical function relating psychological duration to clock time in order to explore timing accuracy. What will be termed here the bias approach looks at deviations of estimates from clock time, i.e. accuracy, as a function of participant groups and experimental conditions. As will be discussed shortly, deviation measures of accuracy bring particular analytic problems that have not been fully explicated in relation to between group effects, such as depression.

This study investigates the effect of mood state and judgment method on direct time judgments, involving durations from 2 s to 65 s, with 5 time categories, termed ‘timecats’ in each experimental condition Two methods of direct judgment are used: estimation and production, with the term ‘judgment’ used here to refer to either method. The term *estimation* is used when an interval, often bounded by auditory signals, is presented to participants, who then give a verbal *estimate* of the duration of that interval; or *production*, is used when participants *produce* an experimenter specified duration. These judgments can be either *absolute* or *relative*. For absolute judgments, participants’ estimations are in seconds and their production is of a duration specified by experimenter in seconds. Relative judgments are always made relative to some standard interval, with no reference to clock time. In the simplest form of estimation, participants are presented with a *fixed* standard at the beginning of a session and told its value is ‘100’, for example. They then give a number for each of a series of presented intervals such that twice as long as the standard is ‘200’ and half as long is ‘50’, etc. Production starts with the same fixed standard interval and participants have to produce intervals that correspond to numbers such as ‘200’, ‘50’, etc. A more complex, but potentially less biased approach uses multiple different standards [9].

The study as reported here had two main aims. The first was to establish whether a depressive realism effects is present for time estimation so one focus is on the effect of mood on time estimation. The second aim was to evaluate the relation between estimation and production judgments. Thus the second focus is on assessing classes of model, as one posited implication of internal clock or pacemaker models is that there will be a negative correlation between estimation and production. A final third aim was to compare results from the absolute judgment with results from the more complex form of relative judgment described above. In the end this final aim was not realized because nearly half the participants appeared unable to successfully perform the version of the relative task used here. The difficulties with the relative task in no way invalidate the highly reliable results from the absolute task. As a discussion of relative task performance and the relative merits of each method are beyond the scope of this paper and we do not discuss further (see [9] for discussion of the differences between absolute & relative judgment).

### The Psychophysical Function Approach

Psychophysical functions relate psychological sensation, Ψ, to physical intensity, P, where Ψ is expressed numerically on a ratio scale. That is, participants are instructed to assign numbers in such a way that if a physical stimulus, of magnitude P1, is perceived as twice as intense as a stimulus, P2, then the number Ψ1, assigned to P1 should be twice as large as the number, Ψ2, assigned to P2. Since the seminal work of Stevens [10], [11], it is well known that the psychophysical function *approximates* a power law. This has been demonstrated for many prothetic continua by regressing log (Ψ) on log (P) and taking the slope of the regression function as *THE* power law exponent for the relevant modality (equation 1). We considered 4 possible forms for the psychophysical function, as shown in equations 1 to [Disp-formula pone.0071585.e002] [Disp-formula pone.0071585.e003] 4.
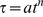
(1)

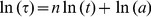
(2)

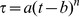
(3)

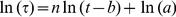
(4)where τ is a psychological judgment of time and t is physical time in seconds.

Marks and Stevens, showed as early as 1968, that equation 4 provided a better fit for several modalities (with the threshold parameter chosen ‘by eye’) [12], [13]. Similarly, Allan (1983) found that equation 2 provided a better fit for her participants for time estimation; and West, Ward and Khosia [14] found that the log form of equation 2 (equation 4) fit best for loudness. Nevertheless the most *prevalent* equation for the psychophysical function is equation 1. It is also well known that features other than the physical magnitude of the stimuli affect the power law exponent. For example, West, Ward and Khosia [14], using equation 4, showed that instructions can change the exponent for loudness; and Marks [15] showed that the frequency of pure tones changed the loudness exponent. There are relatively few such studies for the time modality, and all use equation 1. Glicksohn and his colleagues have investigated the effects of personality traits and attention load on intercepts and slopes for reproduction based psychophysical functions. One study [16] shows a decreased slope for high sensation seekers and another [17] shows no effect on slope, but an increase in intercept for low sensation seekers relative to base line for both low and high overload and for high sensation seekers for high attention load only. Hemmes et al. found lower exponents and higher intercepts when participants engage in an attention demanding secondary task. This literature review shows that psychophysical parameters vary systematically according to condition and participant category. Consequently, studying the effect of manipulating such variables provides a powerful window on the processes underlying time perception.

In order to use the ‘best’ psychophysical function in this study, we evaluated goodness of fit for equations 1–[Disp-formula pone.0071585.e002] [Disp-formula pone.0071585.e003] 4 as follows. Adjusted r^2^ values were obtained separately for all functions for every participant. These adjusted r^2^ values were then converted to Z values to correct for ceiling effects, as r was close to 1 for most functions. Then an ANOVA was conducted with Z as response, mood (depressed, low BDI) as a between factor predictor, and judgment (estimation, production), number of parameters (2, 3) and format (log, power) as repeated measures predictors. The 3 parameter models that include the threshold parameter, **b**, fit better than the 2 parameter models with a substantial effect size, partial eta squared, η^2^ = .57 (η^2^ = .14 is a ‘large’ effect size by convention). The power models fit better than the log models overall, However, post hoc analyses, following up the interaction, shows that this superiority of power models is only present for the 2 parameter models, F (1,38)  = 27.8, p<.0005, η^2^ = .42; and not significant for the 3 parameter models F (1,38)  = 1.0, p = .333. In summary, the best fitting model is the 3-parameter power model, although it was not reliably superior to the 3-parameter log model. A similar re-analysis of previously reported roughness functions [18] gave equation 2 as significantly superior to equation 4. Note that although equation 1 and 3 are equivalent, as are equations 2 and 4, the goodness of fit is not identical, as the loss functions being optimized are different. Consequently, equation 3 is used to estimate parameters for this study.

Using equation 3, complete accuracy is equivalent to **a** = 1, **n** = 1, **b** = 0, when t is in seconds and participants are instructed to provide responses in seconds. So the most accurate groups or participants will produce the intercept parameter **a** and the power law exponent **n** closest to 1 and the threshold parameter **b** closest to 0. The effect of mood and judgment on all three parameters of the psychophysical function is investigated here.

### The Bias Approach

The second approach looks at bias, i.e. the average magnitude of over or under estimation, as the main dependent variable. Most investigations of non-time variables, such as mood, psychopathology, concurrent task, or drugs, use this approach [16], [19], [20], [21], [22], [23]. A variety of methods have been used to get psychological time estimates: including bisection, generalization from a target reinforced duration, categorisation, and the ratio of psychological to time, as well as magnitude estimation and production, see [24] for review. Much of this work is within the framework of the popular scalar expectancy model. This model postulates the following components: an interval clock or pacemaker that is started and stopped by switches when some duration is to be judged (i.e. estimated or produced}; and a gated accumulator [25] that collects ticks in working memory with durations stored in long term reference memory. For estimation, if the clock is running ‘faster’ than real time, then ticks accumulate so quickly that participants will overestimate clock time and time will be judged as passing slower. Such overestimation will also occur if the gate to the accumulator is wide open, because all the participants' attention is focused on the time estimation task [25]. Conversely, in the production task, the participant will keep on timing the interval and accumulating ticks until they have the correct number of ticks copied into working memory, if the clock is running fast or the gate is open wide accumulation will be fast and the target reached quickly, resulting in an underestimation for production. Thus scalar expectancy theory (SET) implies a negative correlation between production and estimation judgments, unless judgments are veridical. Timing may also be influenced by the time taken to turn the switch to the gate on and off, and whether the switch remains open for the whole duration or flickers on and off, both of which may be influenced by different variables than those that influence the clock rate or the gate. See a number of useful sources [26], [27], [28], [29], [30], [31], [32] for descriptions and critiques of the SET model and its variants.

SET is often evaluated by a *linear* regression of subjective time on clock time. It is then assumed that changes in slope imply changes in internal clock speed or equivalently rate of accumulation of ticks [25], [31], [33], [34], [35], [36], [37]. (Obviously, these 2 mechanisms cannot be distinguished empirically). Recently, Mathews has challenged this assumption in an ingenious experiment in which intervals are demarcated by squares of different sizes. If the start and end makers are the same size the slope is different than from when they are different. He argues, convincingly, that since the effect depends on the *end* marker size (which participants do not know in advance), it cannot be influencing what is happening while the clock is ticking and feeding the accumulator. At a more global modelling level it is argued that time estimation must involve at least *two* processes since features that influence the slope and intercept of the *linear* function can be dissociated, e.g. participant anxiety, fear of threat or stimulus intensity or contrast.[1], [2], [4], [5], [38], [39].

Our interest is in mood, i.e. in whether there is a depressive realism effect on time estimation, where people who are mildly depressed are more accurate in time estimation than those who show little evidence of depression through their scores on depression questionnaires such as the Beck Depression Inventory, BDI, [40] (here referred to as low BDI individuals), an effect, so salient in cognitive judgment tasks. Typically the measure used to test for timing accuracy has either been raw deviation τ–t; or the ratio τ/t, as Weber's law and empirical evidence suggests that τ/t (unlike τ–t) does not increase with clock time. (Of course, τ/t only differs from relative difference =  (τ–t)/t = τ/t –1 by a constant, therefore we have chosen to display results for the ratio for simplicity). The measure τ/t has the disadvantage that it is *not* symmetric about the perfect accuracy value τ/t = 1. Symmetry about perfect accuracy is desirable because symmetry treats over and under estimation equivalently. The raw ratio overemphasises over estimation. This is because a subjective estimate twice the true value has τ/t = 1, with difference from perfect accuracy = 2.0–1.0 = 1.0; but a subjective estimate half the true value will have τ/t = .5, with difference from perfect accuracy  = .5–1.0 = −.5. Consequently averaging differences from 1 will have a bias towards overestimation. For this reason we believe that ln(τ/t) is theoretically a preferable measure of accuracy, as it is symmetric about the perfect accuracy value ln(τ/t) = 0. As far as we know, the raw τ–t and ratio τ/t or τ/t–1 measures have not been examined systematically in studies where the main focus was the effect on accuracy of other variables, and ln (τ/t) has not been tried at all.

This study remedies that problem. The effect of the explanatory variables, mood, judgment and time category are investigated using mixed model ANOVA on all three deviation from accuracy measures: raw  = τ–t, ratio  = τ/t, and ln  =  ln(τ /t). This enables not only evaluation of the effect of the explanatory variables, but also evaluation of the mean deviation from perfect accuracy in each combination of conditions. As with the psychophysical functions, the number of individuals showing each pattern of accuracy is also evaluated.

In summary, the major predictor variables are mood, judgment, and time category (timecat). Obviously we expect time judgments to increase with clock time. The psychophysical function question is the form of that increase, as expressed in the parameters, **n, a, b** of equation 3. Our hypothesis is that **n** will not depend on mood or judgment, but that **a** will show a depressive realism effect and be closer to zero for participants who show more evidence of mild depression, here termed the high BDI group; we have no hypotheses about **b**. For the bias approach we predict a depressive realism effect such that mean deviation from accuracy will be smaller for the low BDI group. This hypothesis will be tested separately for the three deviation measures: τ–t, τ/t and ln(τ/t).

## Methods

### Ethics Statement

Ethics approval was granted by the Psychology Ethics Committee under delegated authority from the Ethics Committee of the University of Hertfordshire, to Rachel Msetfi, Protocol Number: PSY/01/07/RM, extended to include Diana Kornbrot and Melvyn Grimwood, Jan 2011.

### Participants

There were 46 students, who participated in this study as a course requirement. Participants were categorized post hoc on the basis of their Beck Depression Inventory scores [BDI: 40] as the: low BDI group, BDI <7, or high BDI group with BDI ≥7). The criterion of BDI ≥7 corresponds to a median split for these recruited participants. This is a lower value than the criterion of BDI ≥9 from the standardisation of the test, [40]; but actually slightly higher than the criterion BDI ≥5 which has been used successfully in some of our previous studies with median split [7], [41]. Thus the high BDI group corresponds to mild depression, sometimes known as dysphoria, often seen in students who are functioning successfully at University. Screening, as described in the results section, reduced the number of participants with data contributing to the final analysis to 39. These comprised 21 low BDI, age (mean 19.9, range 18–28 yrs), BDI (mean 3.4, range 0–6); and 18 high BDI, age (mean 19.9, range 18–26 yrs), BDI (mean 12.5, range 7–26).

### Tasks and Design

All participants completed four counterbalanced conditions comprising two tasks (absolute, A, or relative, R) crossed with two judgment types (estimation, E, or production, P). For absolute estimation, participants judged the duration of presented intervals in seconds, with the following instructions.

“In this task you will be asked to listen to 5 tones of varying lengths. Before you listen to the tones you will be asked to remember a number. When the tone finishes you will be asked to estimate the length of the tone in seconds and remember the number.

Please ask if you have any questions. If you are comfortable to proceed to the experiment press any key.”

For absolute production, they generated intervals specified by the experimenter in seconds with the following instructions.

“In this task you will be asked to generate 5 tones of varying lengths by pressing the space bar to start and finish the tone. Before generating each tone you will be asked to remember a number.

Please ask if you have any questions. If you are comfortable to proceed to the experiment press the space bar.”

All participants judged five durations (timecat) per condition, varying from 2 s to 65 s on a logarithmic scale. Durations are shown in Table 1. All participants received the same stimulus set for production. For estimation, each participant was randomly allocated to one of 4 estimation stimulus sets, produced by jittering from the production set. This was to prevent carry over between conditions and anchor numbers. Within each condition the order of presentation was completely randomised and was different for each participant. Results are reported here *only* for the absolute task, as the relative task was too difficult for several participants. Thus the final design has mood (low BDI, high BDI) as a between group predictor and judgment (estimation, production) and time category (1 to 5) as repeated measures predictors. In all conditions, participants were given a 3-digit number at the start of each trial to be recalled at the end of the trial to prevent counting as a timing strategy. Participants were randomly allocated to one of four counterbalanced orders. Tasks were programmed in Superlab 4.5 on a PC under Windows XP.

**Table 1:** Stimulus durations in seconds.

Timecat		Production		Estimation	
		1	2	3	4
1	5	2	3	4	5
2	10	11	10	12	13
3	18	24	20	16	22
4	34	36	28	39	30
5	65	52	64	55	58

The planned recruitment was 20 low BDI and 20 high BDI participants, with the intention of using all parameters separately as dependent variables. A negative correlation (−.2) between estimation and production was assumed (an estimate based on the known small negative correlation between estimation and production). The final design thus has one between subjects factor, mood, and one within subjects factor judgment. Power was 68% to detect a medium mood effect, f = .25 and 97% to detect a large effect, f = .40.4. Power for the judgment main effect and the mood by judgment interaction was 51% to detect a medium, f = .25, or 89% to detect a large effect, f = .4. Power was calculated using G* [42], [43], [44].

### Procedure

Participants were seated in front of a PC in a quiet cubicle. They then read the general information sheet, signed the consent form, and received a verbal introduction. Each condition started with a screen presenting instructions. Participants initiated the first trial by pressing any key. In the estimation condition the interval to be estimated started and ended with a brief 200 Hz tone. In the production condition the interval started with a brief 200 Hz tone and was terminated by the participant pressing the space bar. There was a short break between conditions. After the fourth condition, participants completed the computerized version of the BDI. Participants were then debriefed and given a sheet including information on services for people feeling depressed.

## Results

### Preliminary Analyses

Inferential tests were carried out at the 95% confidence level, lower and upper 95% confidence levels follow parameter estimates in parentheses. The 46 original participants comprised 24 low BDI and 22 high BDI participants. Psychophysical functions were obtained for each participant for each judgment combination using equation 1 [a linear regression of ln (τ) on ln (t), as is standard practice]. A performance criterion of *R* ^2^_adj_ ≥.90 was used to assess success. Data from 4 out of 24 participants in the depressed group, and 3 out of 24 in the low BDI group, produced *R* ^2^_adj_ <0.90 for at least one condition, or showed disregard of the memory instructions. Data for these participants were excluded from all further analyses and are not discussed further. After this screening process 21 low BDI and 18 high BDI participants remained.

### Psychophysical Function Parameters

Psychophysical parameters **a, n, b,** as defined in equation 3 were estimated for every participant, separately for the estimation and production conditions. This generated 78 psychophysical functions (2 times 21 for low BDI and 2 times 18 for high BDI participants). ANOVAs, with mood (between) and judgment (repeated) as explanatory variables, were then conducted separately for the exponents, **n**, the offset parameters **a**, and the threshold parameters, **b**, as defined in equation 3.

#### Exponent value, n

For the power exponent **n** there were no significant effects of mood, F(1,37)  = 2.17, p = .149; or judgment, F(1,37) =  .90, p = .348; or their interaction, F(1,37)  = 1.86, p = .180, where the mean **n** = .99 (.93, 1.04), SD  = .24, range from .44 to 1.87. Thus the 95% confidence interval for mean **n** spans 1, and so is not statistically different from 1.

There were 39 individual estimation functions and 39 individual production functions. If there is no tendency for **n** to be different from 1, then from 39 functions the predicted frequency of **n** numerically <1 =  predicted frequency of n numerically >1 = 19 or 20. Furthermore, the predicted frequency of **n** *significantly* <1 =  predicted frequency of **n** *significantly* >1∼ = 1. The observed frequency of **n** numerically <1 = 19/39 for estimation (ns); and 29/39 for production, exact p = .001. The observed frequency of **n** significantly <1 = 4/39 for both production and estimation, exact p = .001. There was one function for estimation with **n** significantly >1 as might occur by chance. Hence, although the mean **n** is not significantly <1, at the individual level a significant proportion of functions for production do have **n** numerically less than 1. Furthermore, the proportion of functions with **n**, significantly less than 1 is greater than predicted by chance for both estimation and production.

#### Intercept Parameter, a

By contrast, for the intercept parameter **a**, there was a significant and statistically large mood by judgment interaction, F(1,37) = 7.97, p = .008, η^2^ =  .17, with no reliable main effects. Summary statistics are shown in Table 2. It is noteworthy that for the low BDI group the 95% confidence level of **a** for estimation is above the complete accuracy value of 1, while for production it is below 1. Conversely, for the high BDI group the 95% confidence interval spanned 1 for both estimation and productions. This is a depressive realism effect for the psychophysical function parameter a. The interaction is such that for low BDI participants mean **a** (estimation) > mean **a** (production), F(1, 20) = 4.35, p  = .050, η^2^ = .17; while for depressed participants mean **a** (estimation) < mean **a** (production), F(1, 17) = 4.35, p = .054, η^2^ = .20.

**Table 2:** Summary statistics for offset parameter a as a function of mood and judgment.

Mood	Judgment	Mean	SD	LCL	UCL	Min	Max
Normal	Estimation	2.07	2.09	1.36	2.78	.37	7.12
	Production	1.12	.59	.72	1.52	.09	2.27
Depressed	Estimation	.96	.72	.20	1.73	.05	2.51
	Production	1.65	1.17	1.22	2.08	.01	4.57

Note. LCL is lower 95% confidence level; UCL is upper 95% confidence level.

Table 3 shows the frequency of individual functions, by mood and judgment that are: significantly below 1, below 1, above 1 and significantly above 1. As may be seen, there is no significant asymmetry in any of the four groups. However, what is striking is that the proportion of functions with the **a** value significantly different from 1 is 8/39, exact p = .0003 for estimation and 5/39, exact p = .012, for production.

**Table 3:** Frequency of significance tests for parameter a as a function of mood and judgment.

Mood	Judge	a<1, p<.05	a<1, ns	a>1, ns	a>1, p<.05
Low BDI	Estimation	1	8	10	2
	Production	2	5	12	2
High BDI	Estimation	4	6	7	1
	Production	1	5	12	0

#### Threshold Parameter, b

There were no significant effects on the parameter **b**: for mood F(1,37) = 1.46, p = .234; or for judgment F(1,37) = .28, p = .601; for the mood by judgment interaction, F(1,37) = .50, p = .486. Mean **b** = −.65 (−1.58, .29) is not significantly different from zero, overall or for any sub-group. The range of **b** values was large, −27.1 to 5.0, with a high SD  = 4.5. There are more functions with **b**<0 than with **b**>0, for estimation, 27/39 exact p = .012, but no asymmetry for production 19/39 with **b**<0.

### Accuracy Bias

**Figure 1:**
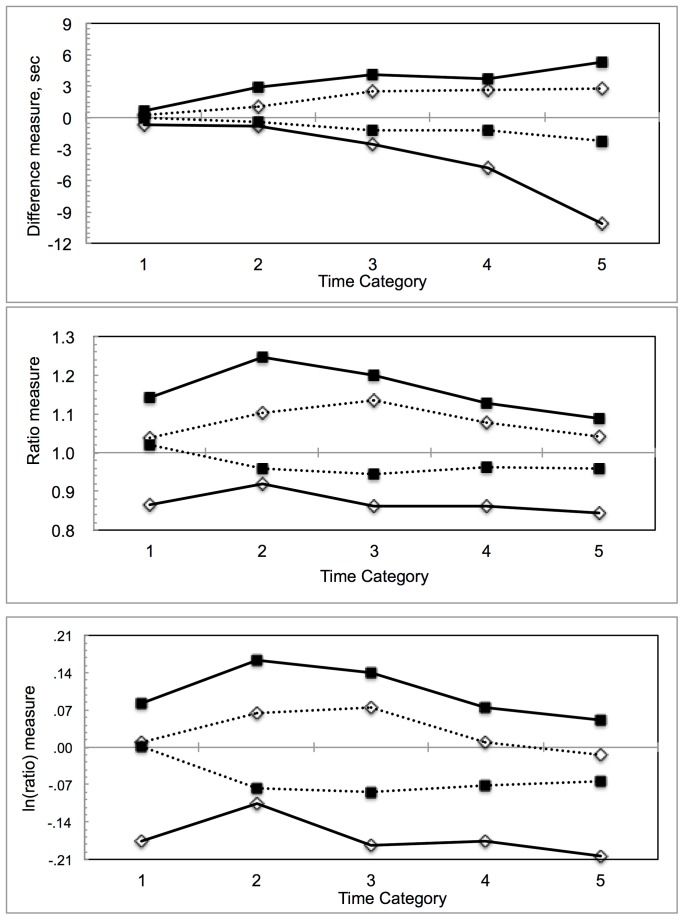
Accuracy measures as a function of time category. Legend: Upper panel, *difference* measure; middle panel *ratio* measure, lower panel, *ln (ratio)* measure. Solid lines, low BDI participants; dashed lines, depressed participants. Filled squares, estimation judgments; open diamonds, production judgments.

**Figure 2:**
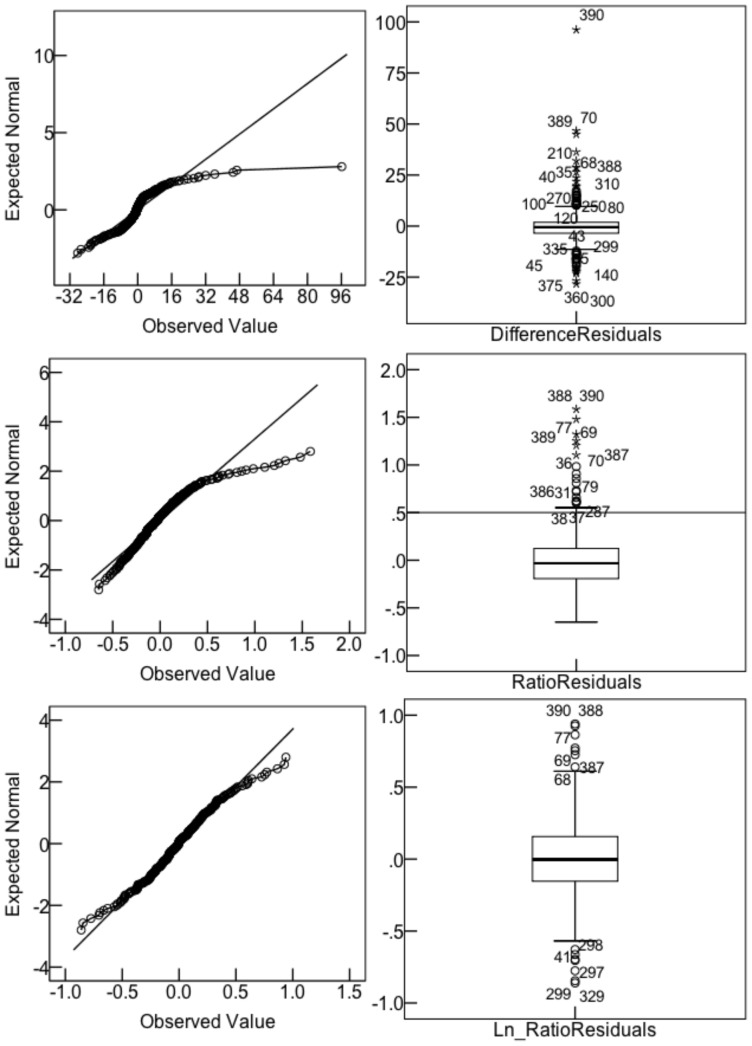
Normality of residuals for three accuracy measures. Legend: Left panels, normal quantile plots; right panels box plots. Top panel, *difference* measure; middle panel, *ratio* measure, bottom panel *ln (ratio)* measure.

Figure 1 shows mean accuracy as a function of mood, judgment and time category for three different accuracy measures: *difference* (τ–t)in top panel, ratio τ/t in middle panel and ln(ratio) ln(τ/t) in bottom panel. Figure 1 suggests both a mood by judgment interaction and some mean deviations from perfect accuracy. So, separate MIXED model ANOVAs were conducted for each of the accuracy measures, with mood as a between groups explanatory variable and judgment and time category as repeated measures explanatory variables (equivalent to repeated measures MANOVA). Normality tests on the residuals showed strong deviation from normality for the difference measure, Kolmogorov-Smirnov (KS)  = .19, p<.0005 and maximum de-trended residual, 2.5 sigma; for the ratio measure, KS  = .10, p<.0005, maximum de-trended residual, 7.0 sigma. However, for the ln(ratio), KS  = .043, p = .082, maximum de-trended residual, 1 sigma. Figure 2 shows box and quantile plots for the residuals for the three accuracy measures, with difference and ratio clearly non-normal ‘by eye’. Normality of residuals is a highly desirable property, since the assumption that the calculated F statistics do indeed have the F distribution depends on this property, without it the null p-values may be simply wrong. These results for the residuals imply that *ln(ratio*) is the preferred measure of accuracy, a new finding. However, results for *difference* and *ratio* measures of accuracy are also reported to compare with earlier work.

#### Mood and judgment effects on accuracy

The ANOVAs provide confidence intervals about means for each accuracy measure, but of course these measures are not directly comparable numerically. Consequently, all measures have been converted into a common metric of percentage over and underestimate, as shown in Table 4, which also shows t- statistics and null p values for the hypothesis that the accuracy measure is statistically significantly different from complete accuracy (0 for difference and ln(ratio), 1 for ratio). There is a striking depressive realism effect in these data. Low BDI participants overestimate by more than 10% on all estimation condition measures, although the departure from accuracy is only significant at the 90% level for ln(ratio) measure, p = .081. They also underestimate significantly by at least 13% on all production condition measures. By contrast, for the high BDI participants no departure from accuracy is greater than 8%, and none is statistically significant, even at the 80% confidence level.

**Table 4:** Mean percentage over or under estimation for all three measures of accuracy.

Measure	Mood	Judgment	Mean	LCL	UCL	t	p
Difference	Low BDI	Estimation	13.3	1.2	25.3	2.23	.038
		Production	−14.3	−27.9	−.7	2.13	.046
	High BDI	Estimation	−4.0	−17.0	9.0	.62	.540
		Production	6.8	−7.9	21.5	.94	.361
Ratio	Low BDI	Estimation	16.0	3.4	28.7	2.58	.018
		Production	−13.0	−25.2	−.9	2.17	.042
	High BDI	Estimation	−3.1	−16.7	10.5	.46	.649
		Production	7.9	−5.3	21.0	1.22	.238
ln(Ratio)	Low BDI	Estimation	10.8	−1.0	24.1	1.84	.081
		Production	−15.5	−23.9	−6.2	3.27	.004
	High BDI	Estimation	−5.7	−16.6	6.5	.98	.339
		Production	3.0	−8.0	15.3	.52	.607

Note. Includes: mean, lower 95% confidence level (LCL), upper 95% confidence level (UCL), t-statistic for departure from accuracy, t, and probability, p, that departure from accuracy is significant.

The ANOVAs showed no main effects for mood (all F(1, 37) <1) or judgment (all F(1,37) <1.8). However, the depressive realism effect shows up for all three measures as significant mood by judgment interactions: for the *difference* measure, F(1,37) = 5.12, p = .029, η^2^ = .12; for the *ratio* measure F(1,37)  = 6.14, p = .0018, η^2^ = .14; for the *ln(ratio)* measure, F(1,37) = 5.85, p = .021, η^2^ = .14.

At the individual level there was no significant asymmetry in the proportion of participants over or under estimating for estimation or production for the low BDI group, or for estimation in the high BDI group for any measure. By contrast, all measures had 17/21 participants underestimating for production, exact p = .0007.

Ln(ratio) and ratio were almost identical in terms of significant departures form accuracy, while difference showed many fewer significant departures. The results are reported for ln(ratio), since this is our preferred measure. For the low BDI group estimation gave 7/21 significant overestimates for, p = <.00005 and 2/21 underestimates, p = .0148; and for production it gave 8/21 significant underestimates, p<.00005 and 2/21 overestimates. For the high BDI group, estimation gave 4/18 underestimates, p = .0001 and 2/18 overestimates; while production gave 3/18 overestimates and 3/18 underestimates p = .0096. Thus the low BDI group shows more significant effects in the predicted direction 14/19, p<.0005; while the high BDI group also shows more significant departures than expected by chance, but with no discernible relation to estimation method.

### Correlations between Estimation and Production Parameters

The correlation between estimation accuracy and production accuracy was calculated for all three accuracy measures, separately for each mood group. All accuracy measures show a strong negative correlation between estimation and production, with all p values <.05 for the high BDI and all p values <.0005 for the low BDI. For the high BDI: r (*difference*)  = −.57, r (*ratio*)  = −.51, r (ln(ratio))  = −.55 . For the low BDI: r (*difference*)  = −.78, r (*ratio*)  = −.72, r (ln(ratio))  = −.77. With this number of participants, the difference between correlations between the groups is not significant. However, power is low: 48% and 93% to detect a medium large effect sizes respectively for the difference between correlations.

At the individual level, it is noteworthy that *no* participant significantly overestimates for both estimation and production, or significantly underestimates for both production and estimation. Significant deviations from accuracy in the same direction for production and estimation would violate assumptions of internal clock timing models.

## Discussion

Psychophysical functions and bias measures were used to assess timing accuracy and between groups differences related to depressed mood. As will be discussed below, data from both types of measure consistently indicated the presence of bias, such that people for whom there was evidence of mild depression (high BDI participants) made time judgments that were generally closer to accuracy than those made by people with low BDI. Below we discuss the results from each measure below, make several methodological recommendations for future work, and then discuss theoretical implications for timing models and depressive realism.

### Time perception measures

#### Psychophysical Functions

The key psychophysical parameter **n** that indexes whether psychological time grows faster, slower or at the same rate as clock time is discussed first. There is no suggestion of any significant effect of either mood or judgment on the power law exponent, **n**. The mean value has **n** = .98 (.93, 1.04) not statistically different from 1. However, using the prevalent two parameter log model gives **n** = .98 (.97, 1.00). Furthermore, the percentage of functions with **n** significantly <1 was 10%, where only 2.5% is predicted by chance. These results are not inconsistent with recent findings of exponents slightly less than unity 45]. The mean exponent is higher than those found by earlier workers, (e.g. [11], [46]). Moreover, Allan's long condition, with times from .4 to 8.1s (most similar to this study) had mean **n** = .91 (.78, 1.04) for the three parameter power formulation. She also found lower values using the prevalent two parameter log formulation, **n** = .84 (.78, .89) [12]. The reason for this difference is unknown.

However, the offset intercept parameter, **a**, does show a depressive realism effect. This finding is consistent with models that suggest that **n** reflects underlying sensory mechanisms, while **a** reflects cognitive factors such as attention that may differ between groups or experimental manipulations. Frustratingly, there is little earlier work that we can consider this finding alongside, as intercepts are often not reported. It is surprising, here, that the threshold parameter, **b**, is not statistically different from zero, since its inclusion substantially improves fit, as described in the introduction. This may be because individual values of **b** can be either positive or negative.

In summary, it appears that both mood and judgment method have at most a small effect on the way subjective time grows with physical time (study was powered to detect a medium effect), the exponent, **n**. By contrast, the mood by judgment interaction on the offset parameter, **a**, is statistically large (η^2^ = .14 is ‘large’ by convention).

#### Bias parameters

All three measures of bias show a depressive realism effect such that the high BDI group was more accurate than the low BDI group.

#### Individual Results

The current study was not designed to have sufficient power to detect differences in patterns of individual results. The findings are nevertheless interesting and suggest that differences in mean values may occur because more participants have values in one direction than another rather than because all participants have small effects in the same direction. Thus mean over estimates in estimation and under estimates in production for the low BDI group may occur because more of these participants have significant results following this pattern. The low BDI group had 9/21 significant departures from accuracy for estimation functions, seven of them over estimates; and 10/21 significant departures from accuracy for production, eight of them under estimates. The high BDI group also had more significant departures from accuracy (6/18) than is expected by chance but evenly split, three over, three under for estimation, and four under and two 2 over for production. Exploring patterns of accuracy amongst individual participants was shown to be useful in this study and deserves more attention.

### Methodological Recommendations

Two main methodological recommendations follow from this work. Firstly, the best fitting psychophysical model should be used to estimate psychophysical parameters. As Allan (1983) pointed out, there is absolutely no excuse for lazily assuming two parameter logarithmic modes, although these may also need to be fitted to compare with earlier results. Good estimates of functional form require judgments of a minimum of 5 different physical values. Its our view, that one gets power per pound (bang per buck) by increasing the number of time intervals to be estimated than by having several replications of each estimate (but we have not yet tested this mathematically).

Secondly, estimates of accuracy, for time estimation at least, should use the *ln (ratio)* measure of accuracy. This is a completely new finding, which should be useful for any modality that uses accuracy as a dependent variable. In this particular study, using measures of accuracy that flagrantly violated assumptions of normally distributed residuals did not change the final conclusion, that a substantial depressive realism effect on time perception exists. However, this is certainly possible and it did change the magnitude of the effect in percentage terms, and magnitudes of effects matter [47]. It is also important to note that if individual psychophysical functions are non-linear, then using mean response across participants, as in an ANOVA of raw data, may lead to biases. As is well known for non-linear models the mean of the individual means is not the same as the mean of the means to each stimulus.

There has been much recent discussion of the shortcomings of psychological research, with social pressures for replication at the forefront of this debate [48], [49], [50], [51]. Meanwhile the effect of using inappropriate statistical tests may have been underestimated, as shown here by the direct comparison of competing measures of accuracy.

### Theoretical Implications

#### Time Perception Frameworks

The negative correlations, between estimation and production parameters, are consistent with a timing mechanism involving the accumulation of ticks on an internal clock. Moreover, these correlations are salient for high BDI as well as low BDI participants in spite of the minimal effects on accuracy. A lack of correlation would have been a challenge to scalar timing. However, there are other explanations for such correlations that have either multiple clocks or no clocks at all, see [24] for a review.

Some workers using the scalar timing framework postulate that variables that interact with physical time must have an effect on the internal clock itself, e.g. [22], [24], [35], [52], or the gating of ticks into the accumulator, e.g. [25], [33], [37]. A slightly different approach, the one used here, is to identify parameters of timing models, and then investigate what variables affect each parameter e.g. [16], [17], [20], [35], [45], [53], [54]. In this study, both mood and judgment affect overall bias, and the intercept but not the slope parameter of the psychophysical function. However, in our view this has no implications either for or against the existence of an internal clock mediating time perception.

#### Depressive Realism

Depressive realism has been demonstrated here to occur in time perception, in addition to the well-documented effects in other domains [3], [55]. The magnitude of the effect is substantial, with 16% overestimates in estimation and 11% underestimation in production for people with no evidence of depression. However, for those for whom depression scores were somewhat elevated (to a similar level to other studies investigating depressive realism see [55] for a review), effects were less that 6% and not significant. The offset parameter, **a**, from the psychophysical functions are consistent with these accuracy biases.

So why are these participants, who score above average on a depression scale more accurate in their timing? The first point to note is that these mildly depressed people who are apparently fully functioning in a University environment are not similar to clinically depressed group, where performance has ***not*** so far been shown to be more accurate than for non-depressed groups (however defined), see [3]. Thus, considering the large body of work which has been carried out on depression and time perception, and time perception in the general population, as well as the current results, we might postulate the existence of a curvilinear relation between mood and time accuracy; whereby states of mild dysphoria are associated with most veridical time perception, and while optimal on one level this may not be most desirable for wellbeing.

The number of emotion or mood related variables that might bring about these effects and influence time perception is large and diverse [1]. So any implications of these findings are inevitably speculative. Both attention and arousal have been suggested as key mediating factors: with higher arousal and greater attention to non-timing stimuli leading to shorter time estimates (a faster clock for those who believe in clocks) e.g. [36], [39]. Thus the high BDI group could be more accurate: (a) because they are in a lower state of arousal; (b) because they are paying more attention to internal timing signals; (c) both (a) and (b); or (d) because debilitatingly high levels of arousal are offset by meticulous attention to their internal clock. In addition it should be noted that the effects of arousal may depend on whether the arousal has negative or positive valence [38]. Based on that study and our other work [3], [8], [38], we speculate that both lower arousal and less attention to external stimuli are mediating mechanisms.

## Summary

Depressive realism is a phenomenon that has been characterised as confined to a small number of specialised situations [55]. However, the results of this study show depressive realism to be strongly evident in timing processes, which are a fundamental aspect of all cognition. Detailed investigations of attention mechanisms, beyond the scope of this report, may be needed to account for the effect. Negative correlations between estimation and production support a ticking clock as a necessary component of human time judgment. The depressive realism effect shows that additional mechanisms, voluntary or involuntary, are needed to explain the full richness of human judgment for time, as for other domains. The choice of the form of the psychophysical function and the measure of accuracy has a profound effect on empirical results and should always be addressed as key components of analyses.
